# Outcomes of Geriatric Hip Fractures Treated with AFFIXUS Hip Fracture Nail

**DOI:** 10.1155/2014/509592

**Published:** 2014-12-18

**Authors:** Ahmed Mabrouk, Mysore Madhusudan, Mohammed Waseem, Steven Kershaw, Jochen Fischer

**Affiliations:** ^1^ST2 Plastic Surgery, St. Andrews Centre for Plastic Surgery and Burns, Broomfield Hospital, Chelmsford, Essex CM1 7ET, UK; ^2^Macclesfield Hospital, Victoria Road, Macclesfield, Cheshire SK10 3BL, UK

## Abstract

Geriatric hip fractures are one of the commonest fractures worldwide. The purpose of this study was to report the outcomes of a series of unstable geriatric hip fractures treated with AFFIXUS hip fracture nail. A retrospective study of 100 unstable geriatric hip fractures treated with AFFIXUS hip fracture nail is presented. The mean follow-up duration was 8 months (range 3–32). Of the patients 83% were female. The average age was 85 years. The fracture was treated by closed reduction and intramedullary fixation. The mean acute hospital stay was 17.6 days. Systemic complications occurred in 29 patients (29%) and local complications in 3 patients (3%) including lag screw cutout in one patient (1%), lag screw backout in one patient (1%), and deep infection in one patient (1%). Mechanical failures and periprosthetic fractures were not observed in our series. Fractures united in all patients. Preinjury activity level was recovered in 78% of the patients. The results of AFFIXUS hip fracture nail were satisfactory in most elderly patients. The unique design of the lag screw and its thread spacing had effectively reduced cut-out rate.

## 1. Introduction

The incidence of hip fractures has been rising in geriatric population in many parts of the world, and the number of hip fractures is expected to reach 512,000 in 2040 [1]. The aim of surgery is to allow early mobilization and prompt return to prefracture activity level [2]. The demand for prompt mobilization with full loading of the affected limb, combined with a desire for the most gentle treatment, becomes increasingly difficult to meet in an ageing patient with advanced osteoporosis [3].

Several clinical and biomechanical studies have analyzed the results of different implants such as the dynamic hip screw (Synthes, USA), the gamma nail (Stryker, Germany), and the proximal femoral nail (Synthes, USA). Those devices have suffered cutout, implant breakage, femoral shaft fracture, and subsequent loss of reduction in the clinical practice [4–8]. AFFIXUS hip fracture nail is a new device introduced in 2011 by DePuy Orthopaedics, Inc., USA. It combines the principle of the compression hip screw with the biomechanical advantages of an intramedullary nail. The major development is the unique design and thread spacing of the lag screw which provides better resistance to cutout.

To our knowledge, there are no studies on the AFFIXUS nail in the literature. The purpose of this study was to report initial results of the AFFIXUS nail in the treatment of geriatric hip fractures.

## 2. Patients and Methods

With approval from our clinical effectiveness department, we conducted a retrospective review of 104 consecutive unstable geriatric hip fractures, treated with AFFIXUS hip fracture nail, at our institution from July 2011 to December 2013. Inclusion criteria were patients aged 60 and above with proximal femur fracture (AO/OTA classification [9] types: 31-A2, 31-A3, 32-A2, and 32-A3). We excluded patients younger than 60 years old with pathological hip fractures and periprosthetic hip fractures. A total of 100 patients were available for outcome analysis in this study. Preoperative variables are listed in Table 1.

**Figure 1:**
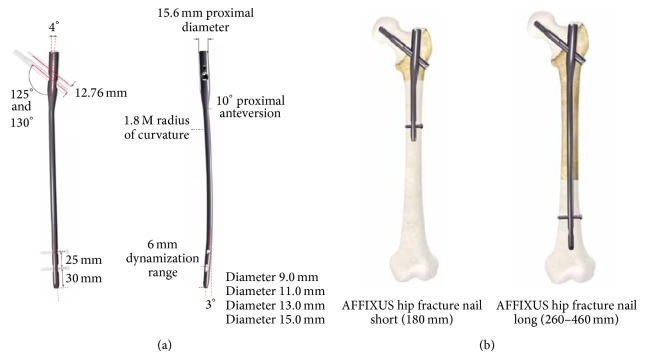
(a) AFFIXUS nail diagram. (b) Short and long AFFIXUS nails.

AFFIXUS nail is available in 2 sizes, short (180 mm) and long (260–460 mm) (Figure 1); the unique thread spacing and lag screw design help to resist displacement and cutout. There is 10° of proximal anteversion built into the nail. The cannulated lag screw measures 10.5 mm in diameter for bone preservation. There is a choice of 125° and 130° neck angles to provide a range of anatomical options. The chamfer on the front distal tip facilitates insertion and decreases risk of stress on the anterior cortex in the distal femur. The 3° distal bend facilitates ease of insertion through the proximal intertrochanteric/subtrochanteric region. There is a preloaded set screw for ease of use and a 5.0 mm antirotation (AR) screw for rotational control. The shouldered lag screw and AR screw help preventing medial screw disengagement.

All surgeries were performed within 48 hours of admission [10]. All fractures were treated by closed reduction under C-arm fluoroscopy control. All operations were performed or supervised by experienced consultant orthopedic surgeon. Any postoperative blood transfusion was recorded. The reduction of head and neck fragment was evaluated by Garden alignment index (GAI) and lag screw position was evaluated by tip apex distance (TAD) [11, 12].

In all cases, antithrombotic prophylaxis was given using low molecular weight heparin and antibiotic prophylaxis was provided. The medical care and rehabilitation protocol was identical, all patients were reviewed by an orthogeriatrician within 48 hours of admission, and patients were mobilized on the first postoperative day. Partial weight bearing as tolerated or restricted weight bearing was allowed according to the surgeon's recommendation on the following day.

The average follow-up period was 8 months (range 3–32 months) with SD (standard deviation) of 7. Clinical and radiographic examinations were performed at the time of admission and at two, four, and eight months postoperatively. We noted any change in the position of the implants and the progress of fracture union. Garden alignment index (GAI) and tip apex distance (TAD) were recorded. Radiographic measurements were performed manually, by two independent observers (Ahmed Mabrouk and Mysore Madhusudan) using a protractor and a ruler to provide values for GAI and TAD.

## 3. Results

The average age of the patients was 85 years (SD 8.1), of which 83% were female. According to AO/OTA classification system [9], 50 cases were type 31-A2 (50%), 46 cases were type 31-A3 (46%), 3 cases were type 32-A2 (3%), and 1 case was type 32-A3 (1%). The long nail (260–460 mm length) was used in 61 cases (61%) and short nail was used in 39 cases (39%). The favored length of the lag screw was 90–105 mm. All distal locking was performed statically by means of one 5 mm screw.

According to Garden alignment index, postoperative X-rays showed a near-anatomical fracture reduction in almost all cases. Implant positioning with a mean TAD 24.9 mm (SD 1.8) was performed in 99 cases (99%). In 95 cases, placement of the lag screw was perceived as “ideal,” in the lower half more to the centre of the femoral neck. Lag screw was placed in the lower third of the femoral neck in 4 cases and in the upper third of the femoral neck in one case.

The mean acute hospital stay was 17.6 days (SD 6.6). Additionally, 27 patients had a rehabilitation period with a mean of 44.2 days (SD 27). During the postoperative period, systemic complications occurred in 29 patients including 11 pneumonia, 8 urinary tract infections, 3 myocardial ischemia, 1 heart failure, 4 postoperative anemia requiring transfusion, 1 hypoproteinaemia, and 1 pulmonary embolism. There were three local complications as reflected in Table 2. We had one deep wound infection that required incision and drainage. Any haematomata of the surgical wound resolved satisfactorily. Superficial infections also resolved favourably once the appropriate antibiotic treatment was instituted.

**Figure 2:**
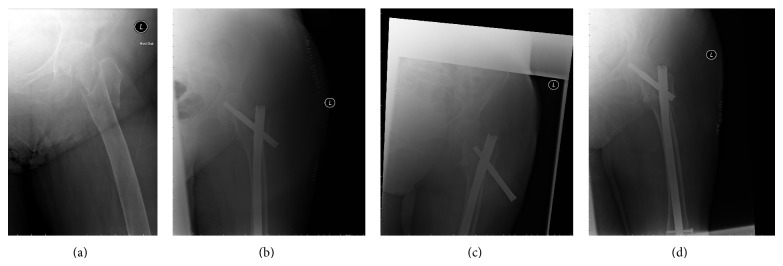
(a) 83-year-old female with proximal femoral fracture (AO/OTA type 31-A3). (b) Immediate postoperative radiograph. (c) Radiograph 15 months after surgery showing the backout. (d) Radiograph after revision of the lag screw.

**Figure 3:**
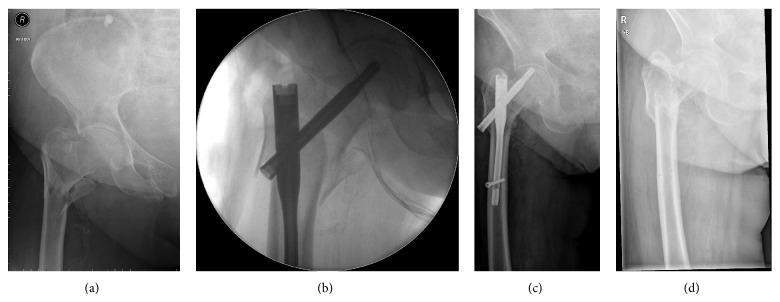
(a) 90-year-old female with proximal femoral fracture (AO/OTA type 31-A2). (b) Intraoperative radiograph anteroposterior view. (c) 5 months after surgery showing the cutout. (d) Immediately after removal of the nail.

**Figure 4:**
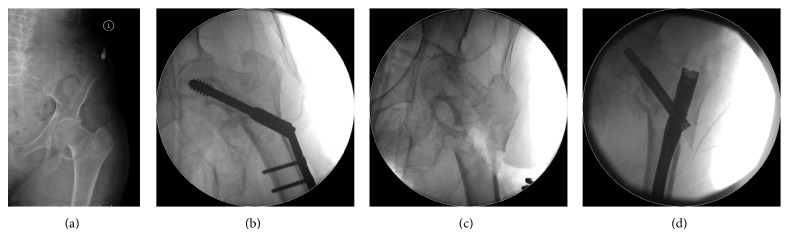
(a) 95-year-old female with proximal femoral fracture (AO/OTA type 31-A3). (b) Intraoperative radiograph showing the DHS. (c) Intraoperative radiograph after removal of the DHS. (d) Intraoperative radiograph showing the revision with AFFIXUS nail.

Any haematomata of the surgical wound resolved satisfactorily. Cases of superficial infection also resolved favourably once the appropriate antibiotic treatment was instituted. Mechanical failures such as bending or breaking of the implant were not seen. Backout was seen in one case, 5 months after surgery for which the lag screw was revised (Figure 2). One case of cutout was observed 15 months after surgery when the fracture had united and AFFIXUS nail was removed (Figure 3). In our series we had one case of on-table revision of DHS into a short AFFIXUS nail (Figure 4), which showed the versatility of AFFIXUS nail indications.

## 4. Discussion

In this study, although the follow-up period was not adequate to measure long-term outcomes, the average 8-month results of AFFIXUS nail fixations were satisfactory. The results showed that AFFIXUS nail provided reliable fixation of hip fractures in elderly patients. The operative procedure for AFFIXUS nail was easily performed, thus reducing blood loss and operative time. In our study, intraoperative variables and systemic complications were similar to those encountered by other authors for different implants in different institutions. However, our local complication rate was much better than those recorded by other authors [5, 13–15]. The overall complication rate requiring further surgery was 3%. The cut-out rate was 1%. The backout rate was 1%. Fracture healing was achieved in all patients. No cases of implant breakage and fatigue were seen during the follow-up period.

Hrubina et al. [13] recorded a total of 39 (11%) specific complications of the dynamic hip screw system in their series. Of these, 44% were intraoperative complications including insufficient reduction, broken tip of a K-wire, faulty technical procedure, and fracture of the distal fragment during surgery, in addition to postoperative complications including “cut-out” phenomenon, avascular necrosis of the femoral head, progression of coxarthrosis, screw breakage, femoral fracture under the plate, pseudarthrosis, and late infection. Hesse and Gächter [5] reported 8% of implant related complications in a series of trochanteric fractures treated with gamma nails. These complications included the following: a short gamma nail needed a conversion to a long gamma nail due to pseudarthrosis or femur fracture at the distal interlocking bolt. Other complications included distal femur fracture through the distal bolt, necessitating a plate osteosynthesis.

In our series, the choice of short and long AFFIXUS nails using a single set of user-friendly instruments made the operative procedure straight forward without intraoperative complications. AFFIXUS nail chamfer on the front distal tip facilitates insertion and decreases risk of stress on the anterior cortex of the distal femur, which reduced the risk of periprosthetic fractures. The design of the lag screw and its thread spacing reduced the incidence of screw cutout. The 2 failure cases included a lag screw backout, which was likely due to inherent instability caused by the Z effect for a proximal femur fracture [16]. The other short AFFIXUS used cutout because the orientation and placement of the lag screw were too superior and TAD was 38 mm.

Baumgaertner et al. [11] described a lower complication rate for implant tips placed close to the subchondral bone of the femoral head as none of the screws with a tip-apex distance of twenty-five millimeters or less cutout, but there was a very strong statistical relationship between an increasing tip-apex distance and cut-out rate, regardless of all other variables related to the fracture. This is similar to our series, as all the lag screws had a mean TAD of twenty-five millimeters. The cut-out case had a TAD of 38 mm.

In conclusion, the results of AFFIXUS hip fracture nail were satisfactory in most elderly patients. The unique design of the lag screw and its thread spacing had effectively reduced the cut-out rate. No mechanical failure was observed in our series.

## Figures and Tables

**Table 1:** Preoperative variables.

Variable	Value
Mean age (Y)	85 (62–102)
Sex (male : female)	(1 : 4)
Side (R : L)	(1 : 1)
Fracture classification (AO/OTA)	
31-A2	50
31-A3	46
32-A2	3
32-A3	1
Mechanism of injury	
Fracture following a mechanical fall	98
Healing insufficiency fracture	1
DHS revision into a nail	1
ASA classification	
1	1
2	18
3	41
4	39

**Table 2:** Postoperative complications.

Complications	Number of cases
Systemic complications	
Pneumonia	11
Myocardial infarction	3
Cardiac failure	1
Urinary tract infection	8
Hypoproteinaemia	1
Deep venous thrombosis	0
Pulmonary embolism	1
Postoperative anaemia required transfusion	4
Local complications	
Deep infection	1
Lag screw cutout	1
Lag screw back out	1
